# Prediction of Passive Torque on Human Shoulder Joint Based on BPANN

**DOI:** 10.1155/2020/8839791

**Published:** 2020-08-26

**Authors:** Shuyang Li, Paolo Dario, Zhibin Song

**Affiliations:** ^1^Key Laboratory of Mechanism Theory and Equipment Design of Ministry of Education, Tianjin University, Tianjin 300072, China; ^2^The BioRobotics Institute, Scuola Superiore Sant'Anna, Polo Sant'Anna Valdera, V.le R. Piaggio 34, Pontedera 56025, Italy

## Abstract

In upper limb rehabilitation training by exploiting robotic devices, the qualitative or quantitative assessment of human active effort is conducive to altering the robot control parameters to offer the patients appropriate assistance, which is considered an effective rehabilitation strategy termed as assist-as-needed. Since active effort of a patient is changeable for the conscious or unconscious behavior, it is considered to be more feasible to determine the distributions of the passive resistance of the patient's joints versus the joint angle in advance, which can be adopted to assess the active behavior of patients combined with the measurement of robotic sensors. However, the overintensive measurements can impose a burden on patients. Accordingly, a prediction method of shoulder joint passive torque based on a Backpropagation neural network (BPANN) was proposed in the present study to expand the passive torque distribution of the shoulder joint of a patient with less measurement data. The experiments recruiting three adult male subjects were conducted, and the results revealed that the BPANN exhibits high prediction accurate for each direction shoulder passive torque. The results revealed that the BPANN can learn the nonlinear relationship between the passive torque and the position of the shoulder joint and can make an accurate prediction without the need to build a force distribution function in advance, making it possible to draw up an assist-as-needed strategy with high accuracy while reducing the measurement burden of patients and physiotherapists.

## 1. Introduction

For patients suffering impaired upper limb function after stroke, adopting rehabilitation robots for rehabilitation exercise can reduce labor burden of therapists, with more accurate measurement of the position and force information in the rehabilitation training. Thus, the quantitative assessment of the patient's health state can be achieved. Recently, the research and application of the rehabilitation robotics has been increasingly common [1, 2]. In therapeutic practice, not all patients lost all their active motion abilities; thus, patients retaining part of the motion abilities can achieve significantly improved training effect of their active participation in the rehabilitation training [3]. As revealed from existing studies, overdose robotic assistance will reduce the patient's active force output and energy consumption in rehabilitation training, and the patient's limbs appear to be “slacking,” probably reducing the efficiency of rehabilitation [4]. Thus, compared with the stiff control strategy that moves the patient's limbs along a desired trajectory in the training process given the patient's active motion ability, the so-called “assist-as-needed” strategy that provides only the minimum assistance required to maximize the patient's active participation can enhance the efficiency of rehabilitation [5].

One of the critical problems of the assist-as-needed rehabilitation strategy refers to the methods to assess the patient's active motion state, which will generate feedback to the robotic therapy devices to modify the control strategy. A common method complies with the surface electromyographic (sEMG), as collected in real time in the rehabilitation training and analyzed online to extract the patient's movement intention [6, 7]. However, applying sEMG to calculate the joint torque usually requires the integration of a complex musculoskeletal model that contains numerous parameters difficult to measure in vivo. Moreover, for patients with neurological impairment due to stroke, the sEMG can be significantly inconsistent with that of the healthy people, and the real movement intention of the mentioned patients may be difficult to successfully extract with the sEMG. There is another type of active motion state assessment method, calculating the patient's active force/moment based on the dynamic model of the human-robot interaction system and the determined value of the robotic device sensor. Obviously, the patients' active force/torque intuitively manifests their motion intention. In fact, the patient's active force/torque is changeable for both of the conscious and unconscious behaviors in the rehabilitation training. Furthermore, unlike the changeable active motion state, a stable nonlinear torque-angle relationship is identified between the passive components of the human joint (e.g., passive resistance of the soft tissues and gravitational torque, as well as the posture of limb for a patients). Thus, a measurement before rehabilitation training to distribute passive components of force/torque of the human shoulder joint is critical to assess active motion states based on dynamic models.

In general, the upper limb is affected by gravitational force/torque, passive resistance force/torque generated by joint biological tissue, active muscle force/torque, and assisted force/torque provided by the rehabilitation device during rehabilitation exercise. In addition, the influence of centrifugal force and inertial force should also be taken into account when the movement speed and acceleration is large. However, considering the safety and comfort of patients, the speed and acceleration of rehabilitation training are usually small; thus, the centrifugal force and inertial force can be neglected. Since the main motion form of the upper limb joints is rotation, the torque is usually concerned rather than the force. Passive torque of the shoulder joint is mainly composed of the gravitational torque and the joint resistance torque. The gravitational torque is determined by inertia parameters such as mass and centroid position. And the joint resistance torque is mainly determined by the viscoelastic characteristics of joint biological tissue. In 1980, the shoulder joint resistance torque of 3 subjects under several simple movement that was measured of upper arm was measured by Engin et al. [8]. The results showed that the magnitude of the shoulder joint resistance torque is obviously different between subjects, but the trend of the torque-angle curves of different subjects is similar. Then, in 1986, Engin et al. measured the shoulder joint resistance torque of the shoulder joint of 10 subjects beyond the active range of motion of each subject, and a statistical database of the torque-angle relationship was formed which can be used in realistic dynamic simulations of human shoulder joint [9]. However, gravitational influence on the shoulder was factored out in their research by making the experimental motion performed only in a horizontal equigravitational plane, which is not easy to realize in actual rehabilitation state. In 2009, an upper limb dynamic model is established by Zhang et al., where each segment of the upper limb was regarded as a rigid body link, and the joint elastic resistance torque and gravitational torque are treated as a whole passive torque [10]. The rate of change of the joint rotation angle according to the dynamic force was defined as joint stiffness. The measurement experiment found that the joint stiffness of the subjects after stroke was significantly increased compared with that of healthy subjects. However, the stiffness value in this study was regarded as a constant value, without considering the change of joint stiffness with a joint rotation angle. In 2019, the passive torque of shoulder joint during external rotation and internal rotation was measured by Wight et al., and the slope of the best-fit line of the torque-angle curve was defined as stiffness [11]. However, in their study, the upper limb rotation was carried out in a fixed plane without considering the distribution of passive torque in other planes. Obtaining the distribution of shoulder passive torque in a wider range is beneficial to evaluating health status and drawing up the rehabilitation strategies, but the measurement of the joint passive torque over the entire joint range of motion may take a long time and make the patients fatigued. Thus, the joint passive torque assessment method with less measurement data can be beneficial.

Since the artificial neural network (ANN) is capable of approximating any rational function without the cognition of the system constitutive model, a prediction method of the shoulder passive torque, as caused both by the gravity and the joint soft tissues, was proposed in the present study based on BPANN, making the expansion of the passive torque distribution of shoulder joint possible.

A passive upper limb abduction experiment was executed with the 7-DoF lightweight collaborative robot KUKA lbr iiwa extensively applied in the human-robot interaction experiments [12]. The position and the force/torque applied to the robot by human upper limb were recorded by the robot sensor in the experimental motion. Subsequently, the kinematic analysis and static force analysis of the upper limb were conducted to calculate the motion and the resistance torque of the shoulder joint. Some of the mentioned angle-torque results were given into a three-layer BPANN as training data. Next, the trained BPANN was adopted to assess the passive torque of the rest joint posture data collected from the identical subject. Afterwards, the torque assessed by BPANN was compared with the torque calculated by static force analysis. The result suggested that the BPANN can accurately assess the spatial distribution of shoulder passive torque.

Results showed that the BPANN assessment method proposed in this study can predict the passive torque of the shoulder joint during the upper limb abduction with high accuracy and make it possible to obtain more passive torque-angle distribution through less measurement data, which is critical to reduce the burden on patients.

## 2. Methods

### 2.1. Subjects

Three healthy male adults were recruited from the identical institution where the experiments of this study were conducted. All subjects were voluntary to participant in the experiment; they were right-handed, with no history of shoulder disease.

### 2.2. Experimental Protocol

**Figure 1:**
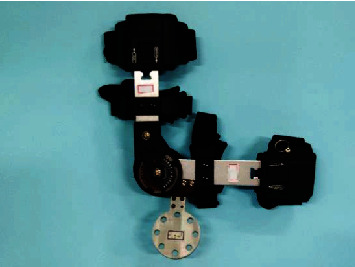
The orthosis of right upper limb.

The subjects were seated at a high chair. The subject's right upper limb was connected to the end tool flange of the robot through the orthosis (Figure 1). To avoid the effect of forearm movement, the elbow joint of the orthosis was locked in a 90° posture, while the shoulder joint movement was not restricted by the orthosis. The subjects were required to maintain the stability of their trunk and avoid the rotation of their upper arm while their right upper limb being dragged by the robot to complete abduction movement in different planes of elevation.

The motion path of the robot was generated by dragging teaching method. In the preliminary stage, the robot was set to a low-stiffness impedance control mode, thus making it possible for the robot to follow the subject's movement. In the dragging stage, the subject was required to move his upper limb along the specified abduction trajectory actively and dragging the compliant robot. The robot recorded the rotation angle of each axis at a frequency of 100 Hz during the dragging step automatically. Subsequently, the trajectory reproduction step was executed in the passive upper limb abduction, in which the robot was set to a impedance control mode with higher stiffness, and the axes angle data recorded in the dragging step was transferred to the controller as the position parameter successively. Thus, the robot can regenerate a similar trajectory of the dragging step. Though the impedance control mode in trajectory reproduction step may lose some positioning accuracy compared with the position control mode for the effect of human upper limb, it can comply with the natural movement trajectory of the upper limbs better, ensuring the safety of the robot and the subjects. Moreover, its compliant properties also helps avoid the sudden change of the joint torque of the robot attributed to the human upper limb as an uncertain load. Accordingly, the impedance control mode is chosen for the passive abduction experiment in the present study. To ensure the safety of rehabilitation training, usually the speed during the motion is slow. Thus, this study only focused on the shoulder resistance performance at low speed, and the speed of each axis of the robot was also limited to 1/10 of its maximum speed.

Before the start of the experiment, the subjects were required to fully warm up the upper limbs. During the passive abduction, the upper limbs of the subjects should not feel being pulled or pushed by the robot obviously. To ensure the stability of the passive torque, all subjects were required to participate in the preexperiment before the formal experiment to determine their muscle relaxation level in the passive abduction. Besides, the sEMG signals of the upper limb muscles related to the active motion were harvested to monitor their muscle activity. As revealed from the results of preexperiment results, the subjects can maintain muscle relaxation during passive exercise. To avoid the interference of the electrode patches and the wires on the subject's motion, no sEMG signal was harvested in the formal passive exercise experiment.

The identical passive abduction trajectory was repeated 2 times in a single experiment. If significant difference is identified between the determined values of the two motion along the identical trajectory, the data will be considered invalid. The position data (axes angle) and force data of the experiment were recorded with the DataRecorder function built in the robot control software; thus, the data of the operation of the robot at the specified frequency (50 Hz in the present study) can be recorded.

### 2.3. Kinematics

The motion and force of real human upper limbs can be significantly complicated, and it is acceptable to make a reasonable simplification when performing kinematic analysis. In the present study, the following assumptions were made:

1. The flexibility of the biological tissue is not considered. The hand takes up a small proportion in the upper limb, and the effect of its motion on the upper limb is negligible. The elbow motion is locked by the orthosis. In the mentioned case, the entire upper limb and the connected orthotics can be considered a whole rigid body for kinematic analysis
2. The shoulder joint is simplified as a ball and socket joint rotating around a fixed point on the human body, and the spatial position of the center of the shoulder joint is assessed with the least-square sphere-fitting method

**Figure 2:**
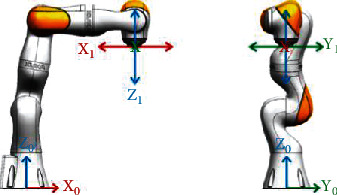
World frame and flange frame of the robot.

Thus, the upper limb is considered a rigid body that rotates around the ball and socket joint at a fixed center. Subsequently, the shoulder joint posture can be calculated from the robot position data recorded by the DataRecorder function. The robot axes angle can be adopted to calculate the position and posture of the flange frame relative to the world coordinate system of the robot by robot forward kinematics. Besides, the world and the flange frames of the robot are illustrated in Figure 2.

**Figure 3:**
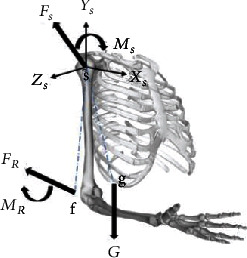
Shoulder joint frame system and upper limb stress.

To quantitatively express the movement of the shoulder joint, the local frame of the shoulder (Figure 3) was built according to the ISB recommendation [13] at its rotation center. In the preliminary stage of the experiment, the subjects altered their sitting posture as guided by the experiment supervisor, thereby making the coronal, sagittal, and vertical axes of their body parallel to the *X*_0_-axis, *Y*_0_-axis, and *Z*_0_-axis of the robot world frame, respectively.

The upper limb and the orthosis are considered a rigid body, and the orthosis is rigidly fixed on the robot flange, so the homogeneous transformation matrix between the robot flange frame and the shoulder frame is considered invariant with upper limb motion. The actual value of the transformation matrix was determined, to be specific, the robot axes angle when the shoulder joint was on its initial posture where shoulder abduction/adduction angle, flexion/extension angle, and internal rotation/external rotation angle were 0° on the whole. Subsequently, the position and posture of the flange frame could be calculated, while the shoulder frame posture was already known (all rotation angle was 0°). Thus, the rotation matrix between the two frames was calculated. Besides, by employing the radius from the shoulder rotation center sphere-fitting, the translation vector between the two frames was calculated. With the rotation matrix and the translation vector, the homogeneous transformation matrix between the robot flange frame and the shoulder joint frame was determined, which can be adopted to calculate the posture of shoulder frame from the robot axes angle as expressed Equation (1), where *T*_*s*_ denotes the homogeneous transformation matrix of shoulder joint frame relative to the robot world frame, *T*_*F*_ represents the homogeneous transformation matrix of robot flange frame calculated by forward kinematics relative to the robot world frame, and _*F*_^*R*^*T* indicates the homogeneous transformation matrix of the shoulder joint frame relative to the robot flange frame.

(1) Ts=TFTFR

Overall, the posture of rigid body is not expressed by the rotation matrix which contains 9 elements directly, whereas it is decomposed into 3 rotation angles in a certain order. The ISB recommended by adopting the YXY order Euler angle to present the shoulder joint (GH joint, actually) posture. However, some existing studies suggested that the YXY sequence Euler angles gives gimbal deadlock problem, and the clinical amplitude coherence is poor [14]. In the present study, the two angles globographic method was adopted to describe the shoulder motion, excluding the rotational effect of the upper arm. The globographic angles were calculated by a landmark point fixed on upper arm, which was taken as the elbow point. The elbow point was obtained by manual measurement.

Though the subjects were required to move their upper limb within a single plane in the passive abduction movement experiments, the elbow joint sampling points did not exhibit the single plane distribution. The mentioned finding is because the movement trajectories were generated by the subjects themselves and because the designated primary movement tended to be accompanied by an unconscious “secondary movement” [15].

**Figure 4:**
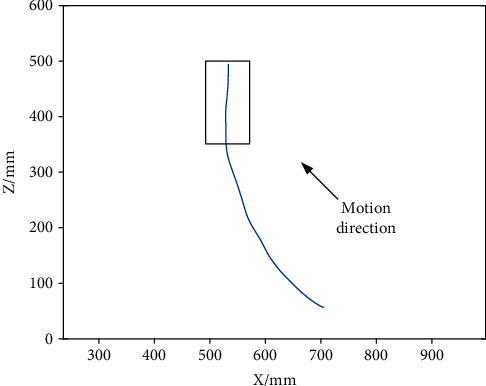
Projection of motion trajectory on XZ plane.

**Figure 5:**
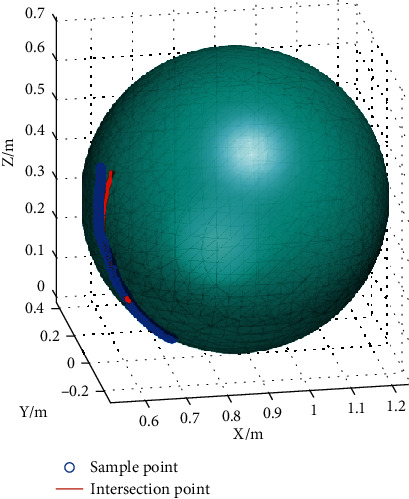
Sphere-fitting results.

It was found in the experiments that the results of sphere fitting were quite different at different stages of a same movement, especially at the end stage. For example, the projection of an abduction trajectory in 0° plane of elevation of subject S1 on the XY plane was shown in Figure 4. The trajectory curve displays a significantly different curvature between the initial and final stages. This is primarily attributed to the translation of the shoulder joint center and for the rigid connection between upper limb and robot that made the rotation of the robot axis more difficult under the larger rotation angle; the effect of the center translation was more obvious than in human natural voluntary movement. Accordingly, for the data of each motion trajectory, the former part was taken for sphere fitting, and the shoulder joint angle was calculated by the intersection point of the line connecting the sample point and the fitting sphere center and the fitting sphere. The fitting sphere with the same trajectory shown in Figure 4 and its corresponding intersection point on the sphere is illustrated in Figure 5.

### 2.4. Static Force Analyses

The passive abduction experiment in the present study was conducted at a slow speed, so the human-robot system is considered quasi-static, and the effect of inertial force was ignored. Moreover, low-speed also reduced the effect of velocity-related viscous part in the passive torque of the shoulder joint.

The force/torque applied on the upper limb under quasi-static is illustrated in Figure 3. The robot applies an assist force *F*_*R*_ on the original point of the flange frame and an assist torque *M*_*R*_ to the upper limb through the orthosis. The shoulder joint generated a resistance force *F*_*s*_ applied on its rotation center and a resistance torque *M*_*s*_. The gravity *G* was applied on the center of mass of the upper limb. Furthermore, the gravity *G* and assist force *F*_*R*_ would generate torque *M*_*G*_ and *M*_*FR*_ at the shoulder rotation center, respectively.

In fact, the rigidly connected robot flange provided constraints of all 6 DoF for the upper limb, and the shoulder joint, which was approximated as a ball and socket joint, generated additional constraints for the upper limb; thus, the upper limb static force system became an overdetermined problem. For this reason, there are infinite sets of solutions for the static equilibrium state of the system in theory. However, an ideal ball and socket joint would only provide force constraints without torque constraints. Likewise, in a specific joint angle, the resistance force *F*_*s*_ of shoulder joint may change following the external force/torque, whereas the resistance torque *M*_*s*_ is relatively stable, primarily determined by the joint tissue characteristics. Moreover, the experimental results revealed that the high repeatability of *M*_*R*_ and *F*_*R*_ of a specific subject in the identical trajectory.

The static equilibrium equation is written in Equation (3).

(2) $$\begin{array}{c} {\text{F}}_{\text{s}}+{\text{F}}_{\text{R}}+\text{G}=0, \end{array}$$

(3) $$\begin{array}{c} M_{s}+M_{R}+M_{G}+M_{FR}=0. \end{array}$$

The robot assist force *F*_*R*_ and assist torque *M*_*R*_ were calculated by the robot joint external torque by Equation (4) derived from the principle of virtual work, where *f* denotes the generalized force of the robot, *τ*_*e*_ represents the external torque on the robot joint calculated by the robot based on its torque sensor measurements with the built-in dynamic model, and *J*^+^ indicates the pseudo-inverse matrix of the 7 × 6 robot Jacobian matrix. The external torque data were smoothed with a moving average filter to reducing the influence of high-frequency noise.

(4) $$\begin{array}{c} f=\left[\begin{array}{c} F_{R}\\ M_{R} \end{array}\right]=J^{+}\tau_{e}. \end{array}$$

The gravity and the center of mass of the orthosis was determined in advance; its effect was removed from the result. The passive torque of shoulder joint *M*_*P*_ can be calculated as Equation (5).

(5) $$\begin{array}{c} M_{P}=M_{s}+M_{G}=-M_{R}-M_{FR}. \end{array}$$

### 2.5. ANN Prediction

**Figure 6:**
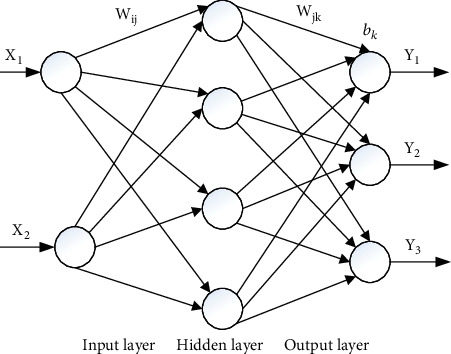
The topological structure of BPANN.

Unlike conventional function fitting methods, the ANN expresses the mapping relationship between input data and output data through the structure and parameters (e.g., weights and biases here) of the layered network. A three-layer feedforward ANN was used here to express the torque-angle relationship of shoulder joint. The number of units of the input layer was two and that of the output layer was three. The number of the hidden units was determined initially by an empirical equation and altered according to assessed effects. After the network structure was determined, the weights and biases of the network could be altered by training. Backpropagation (BP) algorithm is commonly used in ANN training, calculating the gradient of the error with respect to the weights for a given input by propagating error backwards through the network [16]. The topological structure of BPANN is illustrated in Figure 6.

Two globographic angles were selected as the input data and the three components of shoulder passive torque relative to the direction of robot world frame calculated in section 2.4. The activation functions of the hidden and output units were sigmoid. All data were normalized before being transferred to a neural network. The training of the BPANN was carried out in the Neural Network Toolbox of MATLAB. In terms of the training parameters, the maximum number of training epochs was 1000, the performance goal is 0.001 where the performance was measured by the mean square error (MSE) of the network output, and the learning rate is 0.01. The Levenberg-Marquardt optimization was chosen to be the backpropagation algorithm due to its faster training speed.

## 3. Results

### 3.1. Kinematics

**Figure 7:**
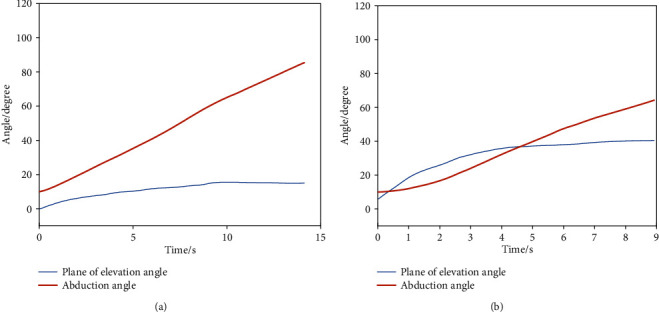
Globographic angle results. (a) Globographic angle of abduction in 0° plane of elevation. (b) Globographic angle of abduction in 30° plane of elevation.

The globographic angle results of the abduction trajectories in 0° plane of elevation and 30° plane of elevation of subject S1 were shown in Figures 7(a) and 7(b), respectively. The angle curves suggested that a secondary movement took place, especially in the moment as presented in Figure 7(b).

### 3.2. Shoulder Passive Torques

**Figure 8:**
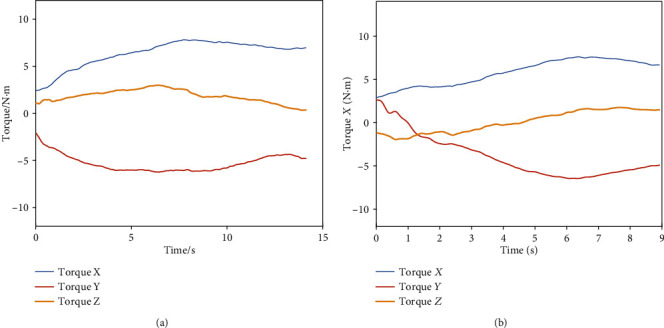
Shoulder joint passive torque results. (a) Passive torque components of abduction in 0° plane of elevation. (b) Passive torque components of abduction in about 30° plane of elevation.

The passive torque calculation results of the two moments in Figure 7 are, respectively, shown in Figures 8(a) and 8(b). It can be seen that the passive torque on the shoulder joint is quite different in different motion trajectories of the same subject.

### 3.3. BPANN Prediction

In the two motions of subject S1 as presented in Figure 7, 1123 groups of angle-torque data were collected. First, 500 groups of data were selected randomly to train the BPANN, and the rest groups of data acted as test set to verify the prediction effect of the network.

**Figure 9:**
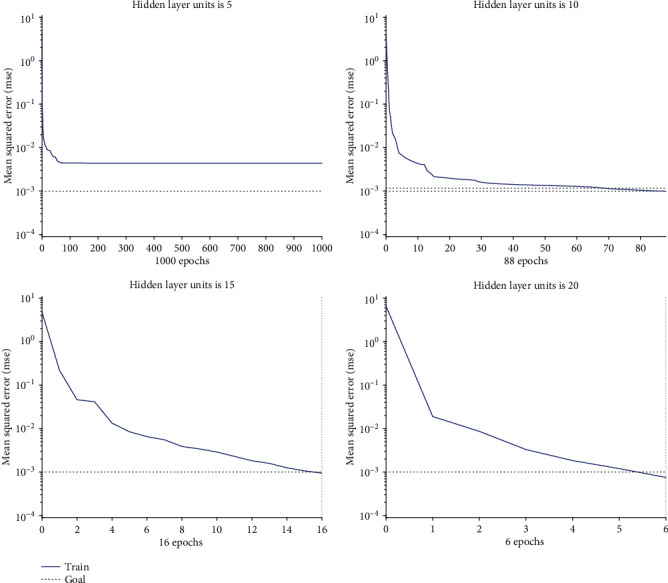
Performance of the BPANN with different hidden layer units.

The number of hidden layer units impacted the prediction effect of the BPANN. Generally, with the increase of the number of neural network layers and hidden layer elements, the nonlinear fitting ability of neural network is enhanced. However, too complicated network structure will increase the calculate complexity and may lead to over-fitting, thus reducing the generalization ability of the BPANN. Therefore, the network structure should be determined according to the prediction effect in practical application. In this paper, the training performance of the network with 5~20 hidden layer units was tested, and the training curves of the networks with different hidden layer units were shown in Figure 9. It can be seen that when the number of the units is small, the network needs more training epochs to achieve the performance goal. Especially when the number of units is very small, the network cannot meet the performance goal, even after more training epochs than 1000. For example, when the number of hidden layer units is 5, the network performance hardly changed with iterative calculation after 84 training epochs, which can not meet the set accuracy goal (0.001). However, although more units can make the network reach the specified accuracy with fewer training epochs, the computational complexity of each epoch is larger. In this paper, the number of the hidden layer units was chosen to be 9, with which the structure of the network will not be too complicated, and at the same time, the accuracy target can be achieved at a relatively fast speed.

**Figure 10:**
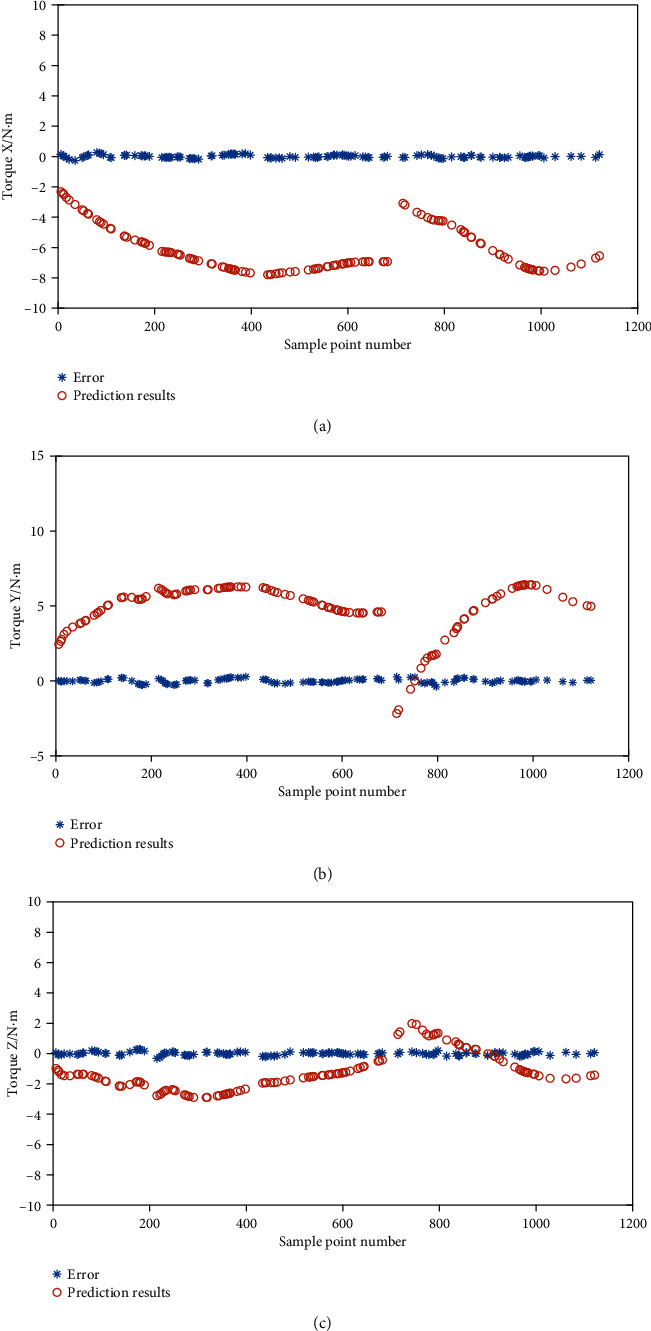
Assessment results and errors in each direction.

The passive torque prediction error of the test set data in each direction was shown in Figure 10. For clarity of illustration, not all sample points in the test set were shown in Figure 10, and one point was taken for every 5 points for plotting. It can be seen that the prediction error was small compared with the magnitude of each passive torque component, and the specific mean absolute value (MAV) and mean square error (MSE) of the passive torque prediction are listed in Table 1. The relative error (RE) is defined as the ratio of the MSE to MAV. The results showed that the BPANN can predict the torque of the shoulder joint with high accuracy.

For subject S2 and S3, similar accurate passive torque perditions can be conducted by BPANN, which was not repeated in this paper for the sake of length. Although the upper limb is often treated as a rigid body link system, in fact, the biological tissue is not rigid and its characteristics, such as inertial parameters and elastic characteristics, will change with the limb motion. Especially for the shoulder joint, its actual motion is coupled by the common motion of the glenohumeral joint, the acromioclavicular joint, the sternoclavicular joint, and the scapulothoracic joint, making the motion and the passive torque on the joint complicated and nonlinear. The coupling motion and passive torque of the shoulder joint has large differences between individuals, but for a specific individual, the relatively stable regularity of shoulder joint motion and passive torque can be found [8, 17]. Considering the BPANN has the ability to learn any nonlinear relation between independent variables and dependent variables, it is suitable for learning the nonlinear relationship between passive moment and joint angle of shoulder joint of a specific individual and expanding the torque-angle distribution. The results showed that the BPANN above can predict the passive torque of a joint angle which is not in the training set with high accuracy.

## 4. Conclusion

In the present study, a shoulder passive torque prediction method based on BPANN was proposed to expand the shoulder passive torque-angle relationship. Experiments were carried out to measure the kinematics and torques on the shoulder joint of 3 healthy subjects, and the measurement data was used as training set and testing set of a three-layer BPANN to test the prediction effect. The results revealed that the BPANN can learn the nonlinear relationship between the passive torque and the position of the shoulder joint and make accurate prediction without the need to build a force distribution function in advance, which is required in conventional curve fitting methods. The prediction method can expand the spatial distribution of the passive torque on shoulder joint with less measurement data, making it possible to draw up an assist-as-needed strategy with high accuracy while reducing the measurement burden of patients and physiotherapists. That is, the BPANN is capable of learning the regularity between the shoulder joint passive torque and the joint position for a specific individual and expand the spatial distribution with less measurement data.

## Figures and Tables

**Table 1:** MAV and MSE of ANN assessment.

	MAV[N·m]	MSE[N·m]	RE
X	6.399	0.093	0.0145
Y	5.172	0.139	0.0269
Z	1.728	0.104	0.0602

## Data Availability

The position data and force/torque data used to support the findings of this study are included within the article.
